# Analytic and Integrative Framework for Understanding Human Sympathetic Arterial Baroreflex Function: Equilibrium Diagram of Arterial Pressure and Plasma Norepinephrine Level

**DOI:** 10.3389/fnins.2021.707345

**Published:** 2021-07-16

**Authors:** Fumiyasu Yamasaki, Takayuki Sato, Kyoko Sato, André Diedrich

**Affiliations:** ^1^Department of Clinical Laboratory, Kochi Medical School, Nankoku, Japan; ^2^Department of Cardiovascular Control, Kochi Medical School, Nankoku, Japan; ^3^Department of Biomedical Engineering, Autonomic Dysfunction Center, Vanderbilt University Medical Center, Vanderbilt University, Nashville, TN, United States

**Keywords:** baroreflex, blood pressure, equilibrium diagram, feedback system, mechanoneural arc, neuromechanical arc, norepinephrine, open-loop gain

## Abstract

**Background:**

The sympathetic arterial baroreflex is a closed-loop feedback system for stabilizing arterial pressure (AP). Identification of unique functions of the closed system in humans is a challenge. Here we propose an analytic and integrative framework for identifying a static operating point and open-loop gain to characterize sympathetic arterial baroreflex in humans.

**Methods and Results:**

An equilibrium diagram with two crossing functions of mechanoneural (MN) and neuromechanical (NM) arcs was analyzed during graded tilt maneuvers in seven healthy subjects. AP and plasma norepinephrine level (PNE), as a surrogate for sympathetic nerve activity, and were recorded after vagal modulation of heart function was blocked by atropine. The MN-arc curve was described as a locus of operating points during –7, 0, 15, and 60° head-up tilting (HUT) on a PNE-AP plane. The NM-arc curve was drawn as a line between operating points before and after ganglionic blockade (trimethaphan, 0.1 mg⋅ml^–1^⋅kg^–1^) during 0° or 15° HUT. Gain values were estimated from the slopes of these functional curves. Finally, an open-loop gain, which is a most important index for performance of arterial baroreflex, was given by a product of the gain values of MN (G_MN_) and NM arcs (G_NM_). Gain values of MN was 8.92 ± 3.07 pg⋅ml^−1^⋅mmHg^−1^; and G_NM_ at 0° and 15° HUT were 0.61 ± 0.08 and 0.36 ± 0.05 mmHg⋅ml⋅pg^–1^, respectively. A postural change from supine to 15° HUT significantly reduced the open-loop gain from 5.62 ± 0.98 to 3.75 ± 0.62. The effects of HUT on the NM arc and open-loop gain seemed to be similar to those of blood loss observed in our previous animal studies.

**Conclusion:**

An equilibrium-diagram analysis contributes to a quantitative and integrative understanding of function of human sympathetic arterial baroreflex.

## Introduction

Arterial baroreflex through sympathetic efferents is the most important negative feedback control system to attenuate the effects of rapid daily perturbations in arterial pressure (AP) (Guyton et al., 1972; Sato et al., 1999b). For example, the fall in AP during a postural change from lying to standing is instantaneously sensed by arterial baroreceptors which initiate an immediate compensatory vasoconstriction and increase in heart rate through activation of efferent sympathetic pathways. Without such compensatory baroreflex response, the simple standing maneuver would cause a fall in AP with the consequence of reduction of brain perfusing and possible loss of consciousness (Ketch et al., 2002; Parikh et al., 2002; Ondrusova et al., 2017).

Guyton et al. (1972) have developed an open-loop analytical approach for characterizing a physiological system with a feedback loop based on new concepts such as the Guyton’s equilibrium diagram for right atrial pressure, venous return, and cardiac output. This diagram enables us to quantitatively and analytically understand how the unique value of the cardiac output is determined by the cardiovascular system. A similar analytical approach for identifying a static operating point of sympathetic arterial baroreflex is needed to understand the mechanism by which AP and sympathetic nerve activity are determined under the closed-loop conditions.

Our previous animal studies with vascular isolation of baroreceptors revealed that the decomposition of the baroreflex loop into mechanoneural (MN) and neuromechanical (NM) arcs allows us to analytically determine the static operating point by equilibrating respective functional curves of the two arcs (Sato et al., 1999b). However, the baroreceptor isolation approach is not applicable to humans. The purpose of the present investigation was to develop a new method and integrative framework for analyzing sympathetic baroreflex control of AP in humans.

## Materials and Methods

### Theoretical Considerations: Coupling of Mechanoneural and Neuromechanical Arcs

**FIGURE 1:**
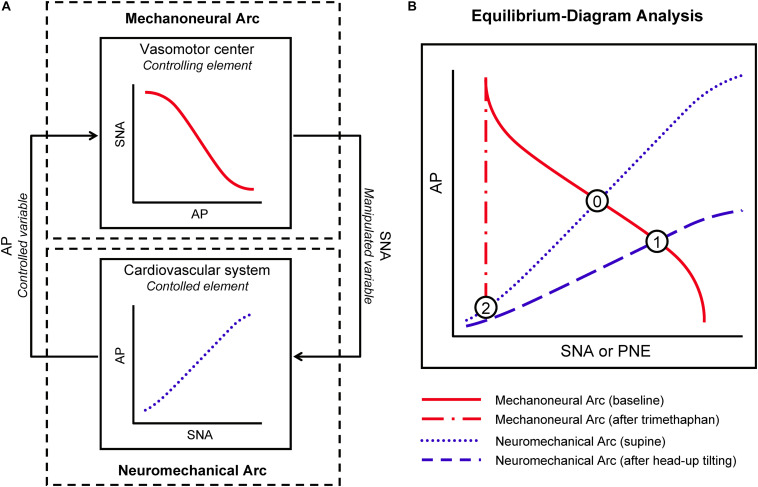
Conceptual scheme for integrative analysis of human sympathetic arterial baroreflex. Sympathetic arterial baroreflex is a feedback control system, which is divided into mechanoneural (MN) and neuromechanical (NM) arcs **(A)**. In the MN arc, its input is a controlled variable, arterial pressure (AP), and its output is a manipulated variable, sympathetic nerve activity (SNA); in the NM arc, its input and output are SNA and AP, respectively. The functional curves of the two arcs can be superimposed on an equilibrium diagram **(B)**. Under closed-loop conditions of the feedback system, its operating point should be a point of intersection between the two curves. PNE, plasma norepinephrine level. See text for detailed explanation of *Points 0,1*, and *2*.

A simplified diagram representing characteristics of the sympathetic arterial baroreflex system is shown in Figure 1A. The vasomotor center modulates sympathetic vasomotor nerve activity (SNA) in response to the changes in AP produced by external disturbance to the cardiovascular system. The changes in AP are immediately sensed by arterial baroreceptors. Changes of efferent SNA with chronotropic and inotropic effects on the heart and vasoconstrictor effects on smooth muscles in peripheral vessels (Hainsworth and Karim, 1976; Robertson et al., 2012; Ichikawa et al., 2019) exert direct influence over AP counteracting the disturbance. As a result, the effect of external disturbance on AP is attenuated by arterial baroreflex. We denote a controlling element of the simplified baroreflex model as a MN arc and a controlled element as a NM arc. In the MN arc, the input is AP, and the output is SNA. In the NM arc, the input is SNA, and the output is AP. Because the variables characterizing the functions of the two arcs are common, we can superimpose the two functional curves and analytically identify the operating point, i.e., the point defined by AP and SNA under the closed-loop conditions of the feedback system, and as an intersection between them on an equilibrium diagram (Figure 1B). The validity of such an equilibrium-diagram analysis for arterial baroreflex has been verified by the previous animal study through a baroreceptor-isolation approach (Sato et al., 1999b).

First, under the closed-loop conditions of the arterial baroreflex system, we draw the functional curve of the MN arc by loading an external disturbance, postural tilting, and on the NM arc. In a supine position, the arterial baroreflex system should operate at an intersection point (*Point 0*) of the two curves on an equilibrium diagram (Figure 1B). During head-up tilt (HUT) the functional curve of the NM arc is shifted downward, and the operating point should move downward and rightward toward higher SNA (*Point 1*).

Trimethaphan is a rapidly acting ganglionic blocking agent and can be used to abolish postganglionic neural activity and to nullify the responsiveness of SNA to AP change (Jordan et al., 1998; Shannon et al., 1998). Therefore, during trimethaphan the operating point should move leftward (SNA reduction) and downward (AP fall) (*Point 2*). Assuming that the PNE can be substituted for SNA, we can redraw the functional curves of the two arcs in the AP-PNE relationship. Therefore, the functional characteristics of the MN and NM arcs are expressed as the PNE response to AP and the AP response to PNE, respectively. Finally, we can identify the functional curve of the MN arc as the line passing through *Points 0* and *1*, and that of the NM arc as the line passing through *Points 0* and *2*. We can also estimate the gain of the MN arc (G_MN_) from the slope of the functional curve of the MN arc against the AP axis, and the gain of the NM arc (G_NM_) from the slope of the NM arc against the PNE axis. Finally, an open-loop gain, G_L_, is given by a product of G_MN_ and G_NM_. G_L_ is a most important index for performance of arterial baroreflex, because the effect of external disturbance should be attenuated to 1/[1 + G_L_] by the closed-loop feedback system (Riggs, 1963; Milhorn, 1966; Kent et al., 1972; Sato et al., 1999b).

### Protocol

To test this concept, a following protocol with tilt and ganglionic blockade was conducted in human volunteers. The research protocol was approved by the ethical review board of Kochi Medical School (Reference number: 2021-4) and followed the Declaration of Helsinki and the ethical standards of the responsible committee on human experimentation.

### Subjects

Seven normal male volunteers aged between 19 and 37 years participated in this study, which was held between November 21, 2001 and November 30, 2001. Their heights and weights were between 1.67 and 1.82 m, and between 57 and 86 kg, respectively.

### Measurement

Each subject was placed on a tilting bed in a quiet and temperature-controlled room (20°C) about 2–3 h after lunch. Surface electrodes were attached to the chest for monitoring of electrocardiogram (ECG). AP was tonometrically measured with a continuous non-invasive blood pressure-monitoring instrument (JENTOW, Colin Electronics, Komaki, Japan) (Sato et al., 1993). The tonometric sensor was attached to the left radial artery. The left upper limb was fixed in shoulder abduction position at 90° of rotation with the use of an arm support. We kept the position of the sensor at the level of the clavicle to monitor the approximate pressure of the subclavian and carotid AP, because arterial baroreceptors sense AP at such regions. An indwelling needle was placed in a forearm vein. Blood was sampled and analyzed for measurement of PNE with an assay. Atropine (0.4 mg⋅kg^–1^) was infused via the forearm venous line to block vagal effects during the protocol (Halmagyi et al., 1969).

### Estimation of Mechanoneural Arc

After a 20 min stabilization period in resting supine position, the posture of the subject was changed to a head-down position of 7°, supine position, HUT position of 15°, and HUT position of 60° every 5 min. The electrical signals of AP, ECG, and angle of the tilting bed were digitized at a rate of 500 Hz by means of analog-to-digital converter (AD12-8(PM); Contec, Tokyo, Japan) and stored. At the end of each 5-min period, blood was sampled for PNE assay.

### Estimation of Neuromechanical Arc

While trimethaphan (Arfonad, Roche) was infused at a rate of 0.1 μg⋅kg^–1^⋅min^–1^, AP and ECG were recorded in a supine position for 5 min. After blood sampling, the posture of the subject was kept in a HUT position of 15° for 5 min under careful monitoring of AP and ECG. At the end of a study protocol, the last blood sampling was made.

### Data Analysis

In each period, AP and heart rate were computed from the data for the last 30 s. AP was calculated by averaging of digitized values. PNE was measured by a high performance liquid chromatography (HLC-8030, Toso, Tokyo, Japan). The relationship between AP and PNE was analyzed for each subject. To characterize the functional curve of the MN arc, we plotted PNE values against AP values measured in four different body positions before trimethaphan infusion. A regression line was fitted to these four points by a least square method, and its slope and AP-axis intercept (AP_MN,0_) was computed. The slope with respect to AP axis was considered as G_MN_. To identify the functional curve of the NM arc in supine position, we plotted supine AP values against PNE values before and after trimethaphan infusion. The line passing through the two points was calculated. The slope with respect to PNE axis, G_NM_(0), was considered as the gain of the NM arc in supine position. The AP-axis intercept of the line, AP_NM,0_(0), was considered as the AP that is generated by the cardiovascular system at null PNE in supine position. To identify the functional curve of the NM arc in 15-degree HUT position, we plotted the AP values in 15-degree HUT position against PNE values before and after trimethaphan infusion, and calculated G_NM_(15) and AP_NM,0_(15). The open-loop gains, G_L_(0) and G_L_(15), at the operating points in supine and 15-degree HUT positions were estimated from the product of G_MN_ and G_NM_(0), and that of G_MN_ and G_NM_(15), respectively.

Paired measurements were analyzed by a Wilcoxon signed-rank test or a Steel multiple-comparison test. Differences were considered significant at *P* < 0.05. Values are expressed as means ± SD.

## Results

### Postural Tilting Tests

**FIGURE 2:**
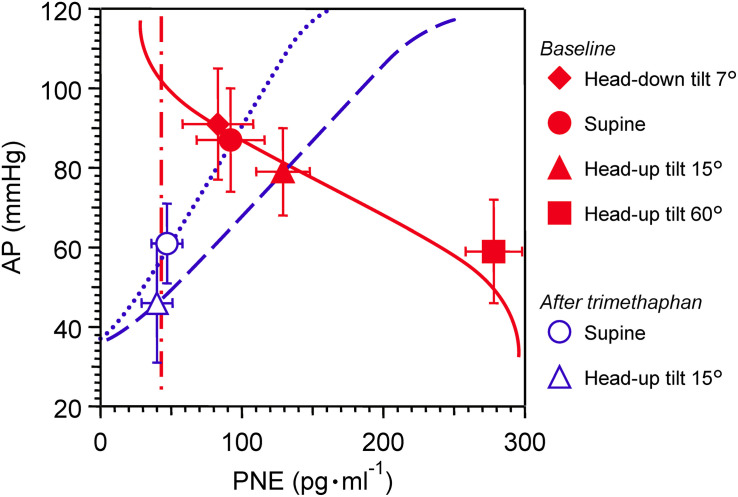
Graph showing effects of postural tilting on arterial pressure (AP), and plasma norepinephrine level (PNE) during baseline and after trimethaphan infusion. Note that a reference level for AP measurement was not the heart but the clavicle; because the controlled or feedback variable of arterial baroreflex should be AP sensed by arterial baroreceptors localized in the wall of the aortic arch and carotid sinus. Values are means ± SD. See text and Table 1 for detailed explanation.

Effects of postural tilting on AP, heart rate, and PNE are shown in Figure 2 and Table 1. Under the baseline condition after atropine injection, head-down tilting increased AP and decreased PNE, and conversely HUT decreased AP and increased PNE. In response to AP changes, PNE exhibited opposite changes.

**TABLE 1:** Effects of postural tilting on arterial pressure (AP), heart rate, and plasma norepinephrine level.

Angle of bed, degrees	–7	0	+15	+60
***Baseline***				
Arterial pressure, mmHg	91 ± 14*	87 ± 13	79 ± 11*	59 ± 13*
Heart rate, beats.min^–1^	96 ± 9	98 ± 13	100 ± 15	118 ± 15*
Norepinephrine, pg.ml^–1^	83 ± 25*	92 ± 24	129 ± 19*	278 ± 42*
***After trimethaphan***				
Arterial pressure, mmHg		61 ± 10†	46 ± 15*†	
Heart rate, beats.min^–1^		89 ± 16†	86 ± 17†	
Norepinephrine, pg.ml^–1^		47 ± 11†	40 ± 11†	

Note that a reference level for measurement of AP was not the heart but the clavicle; because the controlled or feedback variable of arterial baroreflex should be AP sensed by arterial baroreceptors localized in the wall of the aortic arch and carotid sinus. Values are means ± SD. *P < 0.05 from supine position. †*P* < 0.05 from baseline. See text and Figure 2 for detailed explanation.

Trimethaphan significantly lowered both AP and PNE in supine and HUT positions. HUT significantly decreased AP, while it did not increase PNE during trimethaphan infusion. The compensatory responses of PNE to AP changes observed under the baseline condition vanished completely after trimethaphan infusion.

### Estimation of Parameters of Two Arc Curves and Total Loop

Shown in Table 2 are the estimated values for parameters of two arc curves and open loop. G_NM_ and G_L_ were significantly reduced by HUT.

**TABLE 2:** Estimation of arterial pressure offset (AP), gain (G) of mechanoneural (MN) and neuromechanical (NM) arcs, and open-loop gain (G_L_).

Parameter	Estimated value
**MN arc**	
G_MN_, pg⋅ml^–1^⋅mmHg^–1^	8.92 ± 3.07
AP_MN,0_, mmHg	103.0 ± 9.5
Correlation coefficient	–0.96 ± 0.05
**NM arc**	
*Supine position*	
G_NM_(0), mmHg⋅ml⋅pg^–1^	0.61 ± 0.08
AP_NM,0_(0), mmHg	34.1 ± 4.9
*Head-up position of 15 degrees*	
G_NM_(15), mmHg⋅ml⋅pg^–1^	0.36 ± 0.05*
AP_NM,0_(15), mmHg	33.4 ± 8.6
**Open-loop gain**	
G_L_(0)	5.62 ± 0.98
G_L_(15)	3.75 ± 0.62*

Note that values for open-loop gain have no unit. AP is the estimated pressure at zero neural activity. Values are means ± SD. *P < 0.05 from supine position. See section “Materials and Methods” for detailed explanation of definition of parameters.

## Discussion

Here we proposed a model and mechanism for determination of the static operating point of sympathetic arterial baroreflex, showing an integrative framework is applicable to humans. The present results also revealed an important functional index for the regulatory system, open-loop gain, in humans. The open-loop gain, which is referred to as *homeostatic index* (Riggs, 1963; Milhorn, 1966), and is a measure of the ability of the regulatory system to buffer an impact of external disturbance.

### Measurement of Controlled or Feedback Variable

During AP measurement in clinical settings, the arm should be horizontal at the level of the heart as denoted by the midsternal level. The reason for the arm position is that a hydrostatic difference in AP between the brachial artery and the heart should be nullified (Beevers et al., 2001). However, such knowledge should be reconsidered only when we measure AP to evaluate arterial-baroreflex function against orthostatic stress. A standing person with a height of 1.8 m who has AP of 100 mmHg at the level of his heart must have AP of 80–85 mmHg in his baroreceptor areas of aortic-depressor and carotid-sinus nerves because of a difference of 200–250 mmH_2_O in a hydrostatic level (Netea et al., 1998). It should be considered that the controlled or feedback variable of the arterial-baroreflex system should be the AP which is sensed by not the heart but arterial baroreceptors at the aortic arch, brachiocephalic trunk, and carotid sinus (Sato et al., 2002). According to these fundamentals of feedback control theory, we fixed an AP sensor at the level of the clavicle so that we could monitor the approximate value of baroreceptor AP during postural tilting (Yamasaki et al., 2003; Ichikawa et al., 2019).

### Equilibrium-Diagram Analysis With Baroreceptor Isolation Approach

Our previous animal study (Sato et al., 1999b) made a first report on a new analytic framework for understanding sympathetic arterial baroreflex, i.e., equilibrium-diagram analysis of MN, and NM arcs. Using baroreceptor-isolation approach, we could impose any level or waveform of pressure on baroreceptors with a sophisticated servo pump. Under the open-loop conditions, relationship between baroreceptor pressure and SNA and that between SNA and AP were quantitatively measured, and then the operating point of the closed-loop conditions was predicted by equilibrium-diagram analysis. While in real time imposing instantaneous AP on vascularly isolated baroreceptors, we observed the operating point of the closed-loop conditions. Agreement between analytically predicted and actually observed operating points of the closed-loop conditions validated equilibrium-diagram analysis for sympathetic arterial baroreflex.

Our previous study with modeling and simulation revealed the effect of an external disturbance, loss of blood, on MN and NM arcs. The loss of blood volume in the range of 0.5-2% of body weight reduced G_NM_ and AP_NM,0_ dependently on its severity, while it did not affect any parameter of the MN arc. A graphical analysis with equilibrium diagram of MN and NM arcs helps us to understand a mechanism for a shift of the operating point after external disturbance to the circulatory system under the closed-loop or open-loop conditions of arterial baroreflex.

### Equilibrium-Diagram Analysis in Humans

Although many earlier studies of arterial baroreflex elucidated its particular features, such as baroreceptor transduction properties (Sato et al., 1998), central mechanisms (Harada et al., 1993), and effector organ contributions in animals (Shoukas and Sagawa, 1973; Hainsworth and Karim, 1976) and humans (Jordan et al., 1998; Shannon et al., 1998), and few efforts have been made to elucidate an overall behavior of human arterial baroreflex. Accumulation of detailed knowledge of fragmentary components did not allow us to integratively understand how the arterial baroreflex is capable of attenuating the effect of external perturbation on human AP.

**FIGURE 3:**
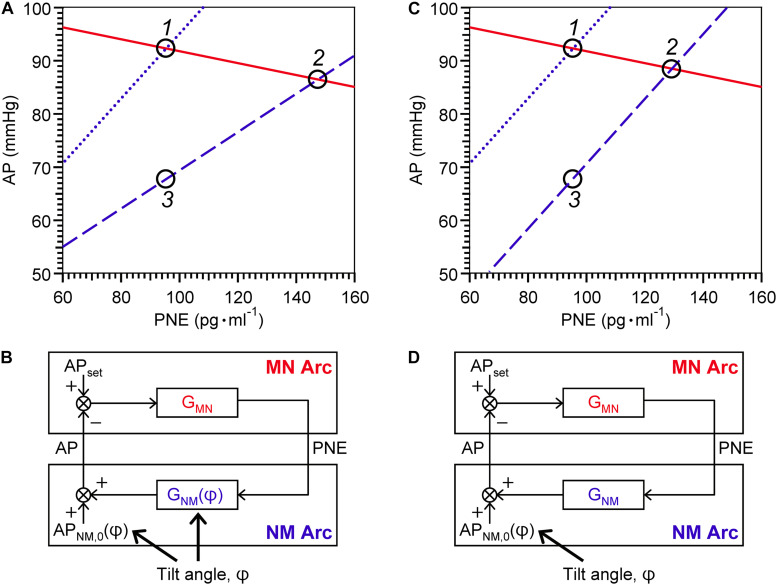
Graphical representation of physiological meanings of gain factors of MN and NM arcs and a loop **(A,C)** against 15° head-up tilting (HUT), and block diagrams of sympathetic arterial baroreflex with **(B)** and without **(D)** a tilt-induced effect on the NM-arc gain. **(A)** The MN- (solid line), supine NM- (dotted line), and HUT NM- (dashed line) are reproduced with its average parameters summarized in Table 2. For a line of the MN arc, AP = – 1/G_MN_ PNE + AP_MN,0_; for a line of the NM arc in supine position, AP = G_NM_(0) PNE + AP_NM,0_(0); and for a line of the NM arc in 15° HUT, AP = G_NM_(15) PNE + AP_NM,0_(15). **(C)** The MN- and supine NM-arc curves are identical with **(A)** while the HUT NM-arc curve is assumed to move down in parallel with the supine NM-arc curve. Using equilibrium diagrams **(A,C)**, we can graphically explain how sympathetic arterial baroreflex attenuates the effect of external disturbance on the circulatory system in humans. During baseline condition, the operating point moves from *Point 1* to *Point 2* along the functional curve of the MN arc in response to changes in tilt angle **(A)**. The difference in AP before and after 15° HUT is estimated to be 6 mmHg. However, if the MN arc does not respond to AP change at all, the operating point should move down vertically from *Point 1* to *Point 3*, and thus the AP difference before and after tilting is assumed to reach 24 mmHg. Consequently, the effect of 15° HUT on AP is considered to be attenuated to 1/4 by sympathetic arterial baroreflex. If this attenuation ratio is used for estimation of G_L_, it is calculated to be 3 according to the following formula: G_L_ = 1/(attenuation ratio) – 1. However, the estimated value for G_L_ is not consistent with G_L_(0). The reason for this inconsistency is that HUT affects G_NM_ as well as AP_NM,0_. If G_NM_ were independent of tilt angle, the NM-arc curve in 15° HUT position would become parallel to that in supine position **(C)**, and then the AP difference between *Point 1* and *Point 2* before and after tilting is assumed to be less than 4 mmHg during baseline condition. Therefore, the G_L_ estimated from the attenuation ratio should exactly coincide with the G_L_(0). AP, arterial pressure; PNE, plasma norepinephrine level; AP_set_, set-point value for AP; AP_NM,0_, AP that is generated by the cardiovascular system at null PNE; G_MN_, gain for MN arc; G_NM_, gain for NM arc; φ, tilt angle. See text for detailed explanation.

Shown in Figure 3A is a representative equilibrium diagram of human MN and NM arcs for understanding of the operating point of sympathetic baroreflex control of AP. Each arc curve is reproduced with its average parameters summarized in Table 2. The effects on the NM arc of HUT in humans seem to be very similar to those of blood loss in animals (Sato et al., 1999b). This similarity would result from a similarity in hemodynamic effects between the two types of external disturbances (Guyton et al., 1972; Yamasaki et al., 2006). Therefore, we suppose that HUT reduces cardiac preload and cardiac output (Critchley et al., 1997) and subsequently decreases G_NM_, and AP_NM,0_ dependently on its angle. In terms of systems physiology, a block diagram of human sympathetic baroreflex control of AP against postural tilting can be drawn as shown in Figure 3B. Taken together, an imposition of the external disturbance, postural tilting, on the circulatory system is considered to alter G_NM_ and AP_NM,0_, and thus these parameters are regarded as functions of tilt angle φ. Therefore, the functional curve of the NM arc is expressed as follows: AP = G_NM_(φ) PNE + AP_NM,0_(φ).

Using the equilibrium diagram, we can graphically explain how sympathetic arterial baroreflex attenuates the effect of external disturbance on the circulatory system in humans. As illustrated in Figure 3A, the operating point moves from *Point 1* to *Point 2* along the functional curve of the MN arc in response to changes in tilt angle. The difference in AP before and after HUT with angle of 15° is estimated to be 6 mmHg during baseline condition. However, if the MN arc does not respond to AP change at all, the operating point should move down vertically from *Point 1* to *Point 3*, and thus the AP difference before and after tilting is assumed to reach 24 mmHg. Consequently, the effect of 15-degree HUT on AP is considered to be attenuated to 1/4 by sympathetic arterial baroreflex. If this attenuation ratio is used for estimation of G_L_, it should be equal to three according to the following formula: G_L_ = 1/(attenuation ratio)–1 (Kent et al., 1972; Sato et al., 1999b). However, the estimated value for G_L_ is not consistent with G_L_(0). The reason for this inconsistency is that HUT affects G_NM_ as well as AP_NM,0_. If G_NM_ were independent of tilt angle (Figure 3C), the NM-arc curve in 15-degree HUT position would become parallel to that in supine position, and then the AP difference between *Point 1* and *Point 2* before and after tilting is assumed to be less than 4 mmHg during baseline condition. Therefore, the G_L_ estimated from the attenuation ratio should exactly coincide with the G_L_(0). Even though a block diagram similar to Figure 3D is well known as an explanation for function of arterial baroreflex with postural tilt-induced perturbation (Milhorn, 1966; Kamiya et al., 2014), it should be repeatedly emphasized that postural tilting should affect not only AP_NM,0_ but also G_NM_ as an external disturbance to the cardiovascular system (Figure 3B). The present results obtained by the equilibrium-diagram analysis would contribute to an integrative understanding of physiology of human sympathetic arterial baroreflex and pathophysiology of supine hypertension with orthostatic hypotension (Ketch et al., 2002; Parikh et al., 2002).

As proposed by our previous study with animals (Sato et al., 1999b), AP_MN,0_ is assumed to be an approximation to a set-point value for a controlled variable of the feedback control system. When AP becomes higher than the set-point value, there is no response of PNE to AP. An integrative and analytic framework with the equilibrium diagram enables us to graphically understand the function of each component and variable of arterial baroreflex.

### Open-Loop Gain

Many investigators have estimated the open-loop gain of sympathetic baroreflex control of AP by perfusing vascularly isolated areas such as one carotid sinus, both carotid sinuses, or the aortic arch at various pressures while measuring AP changes in animals. The ratio of AP change to baroreceptor pressure change, i.e., open-loop gain, was reported to be between 1.0 and 3.5 (Kent et al., 1972; Shoukas and Sagawa, 1973; McRitchie et al., 1976; Burattini et al., 1994; Sato et al., 1999a; Sunagawa et al., 2001). However, such an invasive approach is not applicable to humans, and thus open-loop gain of human baroreflex control of AP has been not yet clarified. Although baroreflex sensitivity or cardiac baroreflex gain is usually used as an index for human baroreflex function (Cooper and Hainsworth, 2001; Ogoh et al., 2006; Fisher et al., 2007; Horsman et al., 2013; Lund et al., 2018; Shahzad et al., 2018), such an index is not an effective surrogate for *homeostatic index* of arterial baroreflex (Riggs, 1963; Milhorn, 1966). To our best knowledge, this is the first study to present an equilibrium-diagram analysis of human sympathetic arterial baroreflex. Using this framework, we can outline the MN and NM arcs and estimate the open-loop gain.

### Limitations

Our previous study with animals revealed an entire picture of the MN and NM arcs with sigmoidal shape by altering a sufficiently wide range of baroreceptor pressure (Sato et al., 1999b). The present human study, on the other hand, could not draw overall characteristics of the two arcs because it is quite difficult to make such a wide range of change in AP. As shown previously, however, the functional curves of the two arcs appeared to be linear in a limited range near baseline operating points of sympathetic arterial baroreflex.

Although PNE was used as an index of sympathetic nerve activity in the present study, PNE is considered to be determined by a difference between the norepinephrine spillover rate from postganglionic sympathetic nerve endings to plasma and the norepinephrine clearance (Esler et al., 1982). Therefore, the interpretation of PNE data, e.g., interindividual comparison of the gain of the MN or NM arc alone, should be made carefully; while, on the contrary, the estimated value for open-loop gain, which has no unit of measurement, has a physiological meaning even when being compared among individuals.

In this study, data were obtained from a small number of healthy and young volunteers. Therefore, we could not conclude that the parameters of the MN and NM arcs shown here are representative of a normal population. Further studies with a large number of healthy or diseased people at different ages are needed for an overall understanding of baroreflex function or dysfunction (Jordan et al., 1998; Shannon et al., 1998).

## Conclusion

In the present study, an equilibrium-diagram analysis for functional curves of MN and NM arcs of human sympathetic baroreflex revealed the mechanism by which AP is stably controlled against postural tilting. Such an analytical framework would help a quantitative and integrative understanding of pathophysiology of arterial baroreflex dysfunction and failure.

## Data Availability Statement

The datasets generated for this study are available on request to the corresponding author FY, yamasaki-f@kochi-u.ac.jp.

## Ethics Statement

The studies involving human participants were reviewed and approved by the Ethical Review Board of Kochi Medical School. Written informed consent for participation was not required for this study in accordance with the national legislation and the institutional requirements.

## Author Contributions

TS conceived research. FY and TS designed the experiment. FY, KS, and TS performed data acquisition. FY and KS preprocessed data. FY, TS, and AD analyzed the data, performed statistical analysis, and created figures and tables. FY, TS, and AD interpreted results and drafted the manuscript. All authors read, edited, and approved the manuscript for submission.

## Conflict of Interest

The authors declare that the research was conducted in the absence of any commercial or financial relationships that could be construed as a potential conflict of interest.
